# Validation of Multiplex Serology detecting human herpesviruses 1-5

**DOI:** 10.1371/journal.pone.0209379

**Published:** 2018-12-27

**Authors:** Nicole Brenner, Alexander J. Mentzer, Julia Butt, Angelika Michel, Kristina Prager, Johannes Brozy, Benedikt Weißbrich, Allison E. Aiello, Helen C. S. Meier, Judy Breuer, Rachael Almond, Naomi Allen, Michael Pawlita, Tim Waterboer

**Affiliations:** 1 Infections and Cancer Epidemiology, German Cancer Research Center, Heidelberg, Germany; 2 Faculty of Biosciences, Heidelberg University, Heidelberg, Germany; 3 The Wellcome Centre for Human Genetics, University of Oxford, Oxford, United Kingdom; 4 Big Data Institute, Li Ka Shing Centre for Health Information and Discovery, University of Oxford, Oxford, United Kingdom; 5 Institute of Virology and Immunobiology, Julius-Maximilians-Universität Würzburg, Würzburg, Germany; 6 Department of Epidemiology, Gillings School of Global Public Health and Carolina Population Center, Chapel Hill, North Carolina, United States of America; 7 Joseph J. Zilber School of Public Health, University of Wisconsin-Milwaukee, Milwaukee, WI, United States of America; 8 Division of Infection and Immunity, University College London, London, United Kingdom; 9 UK Biobank, Stockport, United Kingdom; 10 Nuffield Department of Population Health, University of Oxford, Oxford, United Kingdom; 11 Molecular Diagnostics of Oncogenic Infections, German Cancer Research Center, Heidelberg, Germany; Rational Vaccines Inc, UNITED STATES

## Abstract

Human herpesviruses (HHV) cause a variety of clinically relevant conditions upon primary infection of typically young and immunocompetent hosts. Both primary infection and reactivation after latency can lead to more severe disease, such as encephalitis, congenital defects and cancer. Infections with HHV are also associated with cardiovascular and neurodegenerative disease. However, most of the associations are based on retrospective case-control analyses and well-powered prospective cohort studies are needed for assessing temporality and causality. To enable comprehensive investigations of HHV-related disease etiology in large prospective population-based cohort studies, we developed HHV Multiplex Serology. This methodology represents a low-cost, high-throughput technology that allows simultaneous measurement of specific antibodies against five HHV species: Herpes simplex viruses 1 and 2, Varicella zoster virus, Epstein-Barr virus, and Cytomegalovirus. The newly developed HHV species-specific (‘Monoplex’) assays were validated against established gold-standard reference assays. The specificity and sensitivity of the HHV species-specific Monoplex Serology assays ranged from 92.3% to 100.0% (median 97.4%) and 91.8% to 98.7% (median 96.6%), respectively. Concordance with reference assays was very high with *kappa* values ranging from 0.86 to 0.96 (median *kappa* 0.93). Multiplexing the Monoplex Serology assays resulted in no loss of performance and allows simultaneous detection of antibodies against the 5 HHV species in a high-throughput manner.

## Introduction

Nine human herpesvirus (HHV) species have been identified, i.e. Herpes simplex viruses 1 (HSV-1, HHV-1) and 2 (HSV-2, HHV-2), Varicella zoster virus (VZV, HHV-3), Epstein-Barr virus (EBV, HHV-4), Cytomegalovirus (CMV, HHV-5), human herpesviruses 6 A and B (HHV-6 A and B), human herpesvirus 7 (HHV-7) and Kaposi’s sarcoma-associated herpesvirus (KSHV, HHV-8). According to genetic and biological properties, such as host cell tropism, the *Herpesviridae* family can be divided into three subfamilies, *alphaherpesvirinae* (HSV-1, HSV-2 and VZV), *betaherpesvirinae* (CMV, HHV-6 A/B, and HHV-7), and *gammaherpesvirinae* (EBV and KSHV) [1]. Upon primary infection, human herpesviruses cause a variety of diseases, such as orolabial herpes and genital herpes (HSV-1, HSV-2), varicella (VZV), infectious mononucleosis (EBV) and exanthema subitum (HHV-6 A/B, HHV-7) [2, 3, 4, 5]. Primary infection may be symptomatic or asymptomatic, depending on the infecting virus and the individual’s condition with respect to age and immunocompetence [2, 5, 6, 7, 8, 9]. All herpesviruses establish lifelong persistence in the infected host and undergo a life cycle with both lytic and latent phases [10]. Reactivation of latent infection may be symptomatic, e.g. in case of VZV reactivation as herpes zoster (i.e. shingles) in middle and older aged people [3]. In rare cases, both primary and latent HHV infection can cause severe disease such as HSV-1 encephalitis [11, 12, 13, 14], congenital CMV infection [15], chronic active Epstein-Barr virus infection [16], and EBV- or KSHV-related cancer [17]. EBV has been classified as Group I human carcinogen by the International Agency for Research on Cancer (IARC) and is causally associated with Hodgkin’s, Burkitt’s and extranodal NK/T-cell lymphomas as well as nasopharyngeal cancer, while KSHV is classified as carcinogenic for Kaposi’s sarcoma and primary effusion lymphoma [17, 18]. In addition, EBV and KSHV have been associated with mucosa-associated lymphoid tissue (MALT) lymphoma and multicentric Castleman’s disease, respectively [17].

As infections by herpesviruses are not reversible and illicit a humoral immune response, species-specific antibodies in serum can be used to detect whether individuals have been infected with HHV over their lifetime. Multiplex Serology is a fluorescent bead-based high-throughput method for simultaneous detection of antibodies against multiple pathogen-specific antigens in one reaction vessel using a very low sample volume [19]. Infectious disease assays have been established on this platform for a wide range of pathogens including human papillomaviruses [19], human polyomaviruses [20], *Helicobacter pylori* [21], hepatitis C virus [22], and *Streptococcus gallolyticus subspecies gallolyticus* [23]. More than 40 antigens enabling simultaneous quantitation of antibodies against a variety of pathogens have been successfully included in Multiplex Serology panels in previous studies [24, 25, 26]. For efficient inclusion into such Multiplex Serology panels, newly developed pathogen-specific assays ideally consist of as few antigens as possible. Here, we report the development and validation of Multiplex Serology for HSV-1, HSV-2, VZV, EBV and CMV comprising 1 to 4 antigens each. Validation was conducted step-wise. First, each individual HHV species-specific assay was validated in monoplex format only comprising the HHV species-specific antigens, further called Monoplex Serology. In a second step, the validated HHV species-specific Monoplex Serology assays were combined and incorporated into a Multiplex Serology panel with various other pathogen-specific assays. Statistical performance of HHV species-specific assays in Multiplex Serology was re-evaluated and found to be maintained in multiplex format.

## Material and methods

### Antigen selection and cloning

Sixteen sequences from HHV proteins were selected as antigens for HHV species-specific antibody measurement (Table 1). Most immunogenic and species-specific regions were chosen according to the literature. Whenever possible, signal peptides and transmembrane regions were excluded from the recombinantly expressed proteins. For VZV antigens and EBNA-1 peptide, selected inserts (Table 1) were assembled into pGEX4T3tag vector (modified from pGEX4T3) via gene synthesis (eurofins Genomics, Ebersberg, Germany) [27]. These constructs were also codon-optimized for expression in *E*. *coli* (Table 1).

**Table 1:** Characteristics of selected HHV antigens.

HHV antigen *(gene)*	function	aa	codon optimized^1^	accession no. Uniprot / DNA template	NCBI reference
HSV-1					
gD *(US6)*	membrane glycoprotein	26–340^2^	-	HSV-1 type 1^3^	NC_001806
gG *(US4)*	membrane glycoprotein	26–189^2^	-		
HSV-2					
gD *(US6)*	membrane glycoprotein	26–339^2^	-	untyped genomic DNA^3^	EU445527.1
mgGunique *(US4)*^*4*^	membrane glycoprotein	344–546	-		Z86099.2
VZV					
gE *(ORF68)*	envelope glycoprotein, cell-to-cell spread	31–134^2^	X	P09259	-
gI *(ORF67)*	envelope glycoprotein, cell-to-cell spread	18–295^2^	X	P09258	-
IE63 *(ORF63)*	transcriptional regulator	1–278	X	P09255	-
EBV					
EBNA-1 trunc *(BKRF1)*	replication, latent viral infection	325–641	-	EBV type 1 cosmid DNA^3^	NC_007605.1
EBNA-1 pep *(BKRF1)*		385–420	X	P03211	-
VCA p18 *(BFRF3)*^4^	capsid protein	1–175	-	M-ABA cosmid DNA^3^	NC_007605.1
ZEBRA *(BZLF1)*^4^	replication activator	1–244	-	EBV type 1 cDNA clone^3^	
EA-D *(BMRF1)*	replication (polymerase accessory subunit)	1–403	-	M-ABA cosmid DNA^3^	
CMV					
pp28 *(UL99)*	capsid protein	1–189	-	genomic DNA, strain Towne^3^	FJ616285.1
pp52 *(UL44)*	DNA binding protein	1–432	-		
pp65 *(UL83)*	tegument protein	1–560	-		
pp150N^5^ *(UL32)*	tegument protein	1–550	-		

^1^VZV and EBNA-1 peptide antigens were obtained via gene synthesis and sequence identity was confirmed by manufacturer

^2^transmembrane domain / signal peptide excluded

^3^templates kindly provided by Prof. Dr. Henri-Jacques Delecluse (HSV-1, EBV EBNA-1 trunc), Prof. Dr. Bertfried Matz (HSV-2), Prof. Dr. Wolfgang Hammerschmidt (EBV ZEBRA), Dr. Georg Bornkamm (EBV EA-D, VCA p18), Dr. Stephan Böhm (CMV)

^4^Upon alignment with NCBI nucleotide squences the following deviations were found. VCA p18: C500A (silent), A512G (silent); EA-D: C54T (silent); mgGunique: A1048G (T→A), A1116G (silent), deletion nt 1276–1278 (deletion aa 431), deletion nt 1365–1406 (deletion aa 458–471); for all other antigens, sequenced nucleotide sequences match the corresponding NCBI reference sequences.

^5^N-terminus; C-terminus not additionally informative (unpublished data)

aa: amino acids

Sequences for all other antigens were derived from genomic DNA (HSV-1, HSV-2, CMV) and cosmid clones (EBV), and used as templates for amplification via PCR. Corresponding primers were designed and subsequently ordered (eurofins Genomics). Sequences for viral antigens were cloned into pGEX4T3tag vector. HHV constructs were amplified in E. coli DH5α, commercially sequenced (eurofins Genomics / GATC Biotech, Konstanz, Germany) and validated against reference sequences from the NCBI nucleotide data base (Table 1).

### Antigen expression

Antigens were expressed in *E*. *coli* strain BL21 as described previously [27]. Briefly, antigens were expressed as Glutathione S-Transferase (GST) fusion proteins (GST-X-tag) in pGEX4T3tag vectors encoding N-terminally for GST and C-terminally for the last eleven amino acids of SV40 large T-antigen (tag). pGEX4T3tag carries the amp^R^ gene for positive selection of transformed bacterial colonies in ampicillin-containing medium. In case of VZV, GST-gI-tag, expression was additionally performed in pDB.GST vector (DNASU, Arizona State University Biodesign Institute, DNASU Plasmid Repository, Tempe, Arizona, USA) encoding for a kanamycin resistance instead of ampicillin for selection of co-transformed colonies (co-expression of gE and gI). In both vectors, transcription of GST fusion proteins is inducible by IPTG (tac promotor).

After bacterial cell lysis, lysates were cleared and stored at -20°C in 50% (v/v) glycerol. Quality control of expressed antigens was performed as described previously [22, 27] and included protein gel electrophoresis followed by Coomassie staining, western blot staining for both C- and N-terminal tags to ensure expression of full length antigens and GST capture ELISA for relative quantitation of the fusion proteins [27].

### Reference panels and reference assays

Human reference sera and details on the gold-standard reference assays are shown in Table 2. The numeration of the reference panels (RP) corresponds to the HHV numbering in Roman numerals (I-V). For CMV, two reference panels tested with different reference assays were available (denoted as reference panels Va and Vb). The reference sera were obtained from the Institute of Virology and Immunobiology of the University of Würzburg (HSV, EBV, CMV), from the Detroit Neighborhood Health Study (DNHS: HSV-2, CMV) and the Zoster Associated Pain (ZAP) and Shingles UK (SUK) studies (VZV). Sera were sent to the DKFZ on dry ice and were stored at -20°C until testing. The serum collections are described in detail below.

**Table 2:** Characteristics of reference serum panels.

HHV	provider	reference panel	n Ref+	n Ref-	reference assay
HSV-1	Dr. B. Weißbrich (Institute of Virology and Immunobiology, University of Würzburg)	I	123	80	Enzygnost anti-HSV IgG (Siemens Healthcare Diagnostics)
HSV-2	Prof. Dr. A. Aiello (University of North Carolina, Gillings School of Global Public Health)	II	61	46	LIAISON HSV-2 Type Specific IgG (DiaSorin)
VZV	Prof. Dr. J. Breuer (University College London)	III	97	83	Time-resolved fluorescence immunoassay (TRFIA)
EBV	Dr. B. Weissbrich	IV	136	65	Enzygnost anti-EBV IgG (Siemens Healthcare Diagnostics GmbH)
CMV	Dr. B. Weissbrich	Va	76	129	Enzygnost anti-CMV IgG (Siemens Healthcare Diagnostics)
CMV	Prof. Dr. A. Aiello	Vb	100	101	ELISA: Stanley Neurovirology Laboratory (John Hopkins University) [34]

n Ref+: number of reference assay positives

n Ref-: number of reference assay negatives

The reference serum panel obtained from the University of Würzburg was composed of two subgroups. Group 1 consisted of serum samples (n = 197; median age 16.9 years (range 0.3–84.6 years); 53% male) received by the viral diagnostic laboratory between 2007 and 2014 for analysis of herpesvirus IgG antibodies. This was part of routine work-up in patients before solid organ transplantation and in patients with malignant diseases before chemotherapy and potentially stem cell transplantation. Group 2 consisted of serum samples (n = 22; median age 1.2 years (range 0.4–25.6 years); 64% male) that were found to be negative for HHV6 IgG antibodies in routine diagnostic testing. With few exceptions, all samples were tested for IgG antibodies against HSV, EBV, and CMV at the viral diagnostic laboratory. The sera were stored at -20°C before shipping to the DKFZ. The use of human serum samples in this study was approved by the ethics committee of the medical faculty at the University of Würzburg. The need for consent was waived by the ethics committee.The DNHS is a longitudinal study of ecologic factors that may influence mental and physical health in an urban setting. DNHS participants are representative of Detroit, Michigan in terms of age, gender, race, income and educational attainment. Participants provided written informed consent for participation and study was approved by the University of Michigan and the University of North Carolina Institutional Review Board (IRB #13–3999) [28]. The reference samples from DNHS were obtained from Wave 1 participants. Frozen serum samples stored at -70°C were shipped on dry ice to the Stanley Neurovirology Laboratory of the John Hopkins University School of Medicine in Baltimore, Maryland to be tested for serum IgG antibodies to CMV and HSV-2 [29]. For each sample, the antibody levels were expressed as ratio of the optical density of a test sample to that of a standard sample assayed in each test run. Individuals were categorized as seronegative if their ratio value was <1.0 and seropositive if ≥1.0.

The VZV reference sera were collected from multiple sources. In the ZAP and SUK study, subjects presenting with acute zoster were followed up for 12 (ZAP) and 6 months (SUK) with serum samples obtained at four time points [30, 31, 32]. Additional sera from VZV positive asymptomatic blood donors were also included in the reference panel [33]. All samples were obtained under the UCLP DNA biobank ethical framework (REC reference: 17/LO/1530). Participants provided written informed consent.

### Monoplex and Multiplex Serology

The reference sera were analyzed for antibodies against selected HHV antigens (Table 1) by species-specific Monoplex and Multiplex Serology, as described previously [19]. Briefly, HHV GST-tag fusion proteins were loaded onto glutathione casein-coated fluorescence-labelled polystyrene beads (COOH-beads xMAP Technology Microspheres, Luminex Corp. Austin, Texas, USA) by *in situ* affinity purification from lysate. Up to 100 bead sets are distinguishable by the Luminex flow cytometer via different ratios of two fluorescent dyes within the polystyrene microspheres. Loading each antigen onto a specific bead set enables simultaneous measurement of antibodies against different antigens within one reaction vessel.

Detection of bound primary antibodies from serum took place with a biotinlyated goat-α-human IgM/IgG/IgA secondary antibody (1:1000, #109-065-064, Jackson Immunoresearch, West Grove, PA, USA) and subsequent incubation with streptavidin-R-phycoerythrin (1:750, PE-Streptavidin Conjugate, MOSS Inc., Pasadena, CA, USA) as reporter dye. Median Fluorescence Intensities (MFI) from at least 100 detected beads per bead set (e.g. antigen) were calculated for each serum. Monoplex Serology was conducted for each HHV species-specific assay in an individual experiment only comprising the species-specific antigens and GST-tag antigen for background subtraction in dilutions 1:100 and 1:1000. Optimal serum dilution was 1:1000 with the exception of VZV (1:100). In addition, performance of HHV Monoplex Serology assays were assessed in multiplex format by combining them with various pathogen-specific Monoplex Serology assays (e.g. human herpes viruses 6–8, human polyomaviruses, human papillomaviruses, human hepatitis B and C viruses) into a Multiplex Serology panel.

### Statistical analysis

The reference sera were tested blinded. The Wellcome Centre for Human Genetics functioned as a trusted third party, and combined the DKFZ testing results with the reference data provided beforehand, thus unblinding the analysis. Each antigen-specific serostatus was determined by applying a cut-off to dichotomize the MFI values into seropositive or seronegative. The final cut-off was determined to result in specificity and sensitivity of at least 85% analogous to Receiver Operating Characteristics analysis. This was achieved by gradually raising a working cut-off from a minimum of 30 MFI (dilution 1:1000) or 50 MFI (dilution 1:100) to optimize specificity and sensitivity. The optimum cut-off was determined to favor specificity, unless a further increase of the cut-off resulted in a disproportional loss in sensitivity. Thus, agreement with the reference assay was maximized. When multiple pathogen-specific antigens were included in the assay, seropositivity against the respective pathogen (denoted as overall pathogen seropositivity) was additionally determined by systematic investigations of antigen combinations. Antigen-specific cut-offs were adapted to optimize agreement with the reference assay if necessary as described above. In addition to sensitivity and specificty, Cohen’s *kappa (k)* statistics to define agreement with the reference assay were calculated and evaluated as follows: 0.01<*k*<0.20: slight agreement, 0.21<*k*<0.40: fair agreement, 0.41<*k*<0.60: moderate agreement, 0.61<*k*<0.80 substantial agreement and 0.81<*k*<0.99: almost perfect agreement [35]. Sensitivity, specificity and *kappa* statistics including 95% Confidence intervals (CI) were calculated using SAS 9.4.

Comparison of the Monoplex and Multiplex Serology performance on the corresponding reference serum panels was conducted by calculating Intraclass Correlation Coefficients (ICCs) using R 3.5.0 package ‘psych’ [36]. ICC(3,1) plus corresponding 95% CI are reported. ICCs were evaluated as follows: 0.01<ICC<0.49: poor reliability, 0.50<ICC<0.74: moderate reliability, 0.75<ICC<0.89: good reliability, 0.90<ICC<1.00: excellent reliability [37].

## Results

### Antigen development

Based on reported immunogenicity, antigen coverage by the reference assays and sequence homology, 2-4 antigens were selected for development and validation of HHV species-specific Monoplex Serology assays (Table 1). Antigens were expressed as recombinant GST-fusion proteins as described previously [27]. To ensure correct protein sequence, parental plasmids were sequenced. For most antigens, perfect agreement with the reference sequence (NCBI nucleotide database) was confirmed (Table 1). Only HSV-2 antigen mgGunique showed non-silent nucleotide sequence variations compared to strain HG52 resulting in one amino acid change, one single amino acid deletion and one 14 amino acid deletion.

### Comparison of HHV species-specific Monoplex Serology assays with reference serostatus

**Fig 1:**
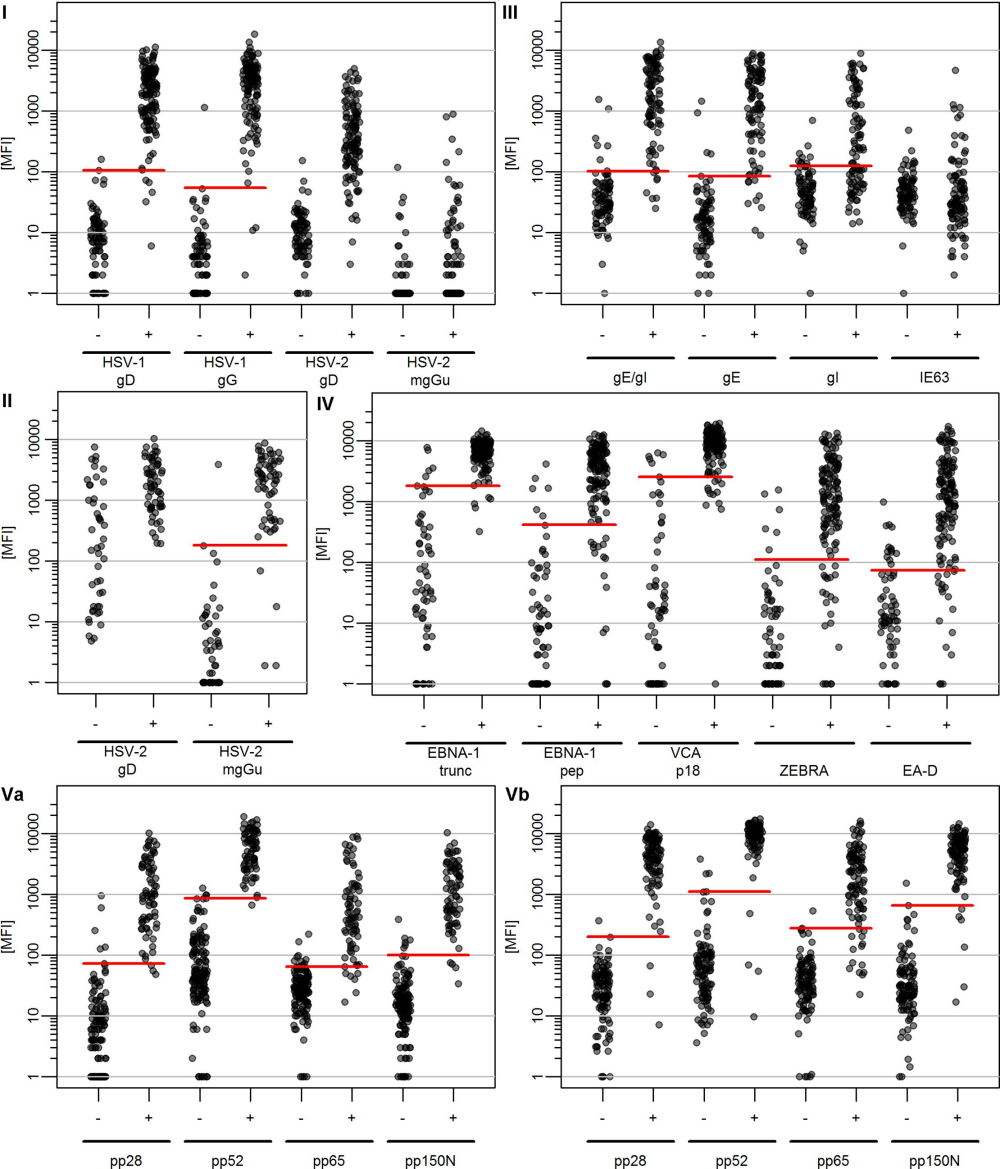
Comparison of quantitative antibody measurements (MFI) with reference serostatus in HHV species-specific Monoplex Serology. I-Vb indicate corresponding reference panels. gE/gI: co-loading of antigens gE and gI onto one bead set red lines: optimized cut-offs for single antigen performance; cut-offs were determined by optimizing specificity and sensitivity. MFI: Median Fluorescence Intensity.

Six reference serum panels (RP I-IV, Va, Vb; Table 2) were analyzed by the corresponding HHV species-specific Monoplex Serology assay. For CMV, two reference panels using different gold-standard assays were available and tested (Va, Vb). Quantitative antibody reactivities (MFI) for each HHV antigen were compared against the corresponding reference serostatus (Fig 1). Where multiple species-specific antigens were included, overall seropositivity in Monoplex Serology was calculated by a combination of the included antigens. Cut-offs were determined by optimizing sensitivity and specificity. The performance characteristics (i.e. specificity, sensitivity and *kappa* statistics) for the Monoplex Serology assays for HHV 1–5 compared with the gold-standard reference assays are shown for each antigen (Table 3) and overall seropositivity (Table 4).

**Table 3:** Single antigen performance compared to corresponding reference panels in Monoplex Serology.

RP	antigen	cut-off^1^ (MFI)	specificity (95% CI)	sensitivity (95% CI)	*kappa* (95% CI)
I	HSV-1				
	gD	106	98.8 (93.2–100)	95.9 (90.8–98.7)	0.94 (0.89–0.99)
	gG	54	98.8 (93.2–100)	97.6 (93.0–99.5)	0.96 (0.92–1.00)
II	HSV-2				
	mgG unqiue	180	97.8 (88.5–100)	93.4 (84.1–98.2)	0.91 (0.83–0.99)
III	VZV				
	gE	85	95.2 (88.1–99.8)	90.7 (83.1–95.7)	0.86 (0.78–0.93)
	gI	124	89.2 (80.4–94.9)	58.8 (48.3–68.7)	0.47 (0.35–0.59)
	gE/gI^2^	101	91.6 (83.4–96.5)	92.8 (85.7–97.1)	0.84 (0.77–0.92)
IV	EBV				
	EBNA-1 trunc	1800	92.3 (83.0–97.5)	96.3 (91.6–98.8)	0.89 (0.82–0.96)
	EBNA-1 peptide	411	90.8 (81.0–96.5)	88.2 (81.6–93.1)	0.76 (0.67–0.85)
	VCA p18	2526	92.3 (83.0–97.5)	91.2 (85.1–95.4)	0.81 (0.73–0.90)
	EA-D	110	83.1 (71.7–91.2)	83.8 (76.5–89.6)	0.64 (0.53–0.75)
	ZEBRA	74	89.2 (79.1–95.6)	86.0 (79.1–91.4)	0.72 (0.62–0.82)
Va	CMV				
	pp28	73	96.1 (91.2–98.7)	96.1 (88.9–99.2)	0.92 (0.86–0.97)
	pp52	854	97.7 (93.4–99.5)	98.7 (92.9–100)	0.96 (0.92–1.00)
	pp65	64	91.5 (85.3–95.7)	92.1 (83.6–97.1)	0.83 (0.75–0.90)
	pp150N	100	95.4 (90.2–98.3)	94.7 (87.1–98.6)	0.90 (0.83–0.96)
Vb	CMV				
	pp28	200	99.0 (94.6–100)	97.0 (91.5–99.4)	0.96 (0.92–1.00)
	pp52	1101	97.0 (91.6–99.4)	96.0 (90.1–98.9)	0.93 (0.88–0.98)
	pp65	276	99.0 (94.6–100)	86.0 (77.6–92.1)	0.85 (0.78–0.92)
	pp150N	655	99.0 (94.6–100)	94.0 (87.4–97.8)	0.93 (0.88–0.98)

^1^ cut-offs determined by optimization of specificity and sensitivity

^2^gE/gI: co-loading of both antigens onto one bead set to simulate heterodimerisation

CI: confidence interval

RP: reference panel

MFI: median fluorescence intensity

**Table 4:** Overall HHV species-specific performance compared to corresponding reference panels in Monoplex Serology.

RP	HHV	antigen	cut-off^1^ (MFI)	criterion	specificity (95% CI)	sensitivity (95% CI)	*kappa* (95% CI)
III	VZV	gE	85	≥ 1+	94.0 (86.5–98.0)	91.8 (84.4–96.4)	0.86 (0.78–0.93)
		gI	160				
IV	EBV	EBNA-1 trunc	1800	≥ 2+	92.3 (83.0–97.5)	97.1 (92.6–99.2)	0.90 (0.83–0.96)
		VCA p18	2526				
		ZEBRA	200				
		EA-D	300				
	EBV	EBNA-1 peptide	600	≥ 2+	92.3 (83.0–97.5)	95.6 (90.6–98.4)	0.88 (0.80–0.95)
		VCA p18	2526				
		ZEBRA	200				
		EA-D	300				
Va	CMV	pp28	73	≥ 2+	96.9 (92.3–99.2)	98.7 (92.9–100)	0.95 (0.90–0.99)
		pp52	854				
		pp150N	200				
Vb	CMV	pp28	200	≥ 2+	100.0 (96.4–100)	96.0 (90.1–98.9)	0.96 (0.92–1.0)
		pp52	1101				
		pp150N	655				

≥ 1+: seropositive against at least one antigen

≥ 2+: seropositive against at least two antigens

CI: confidence interval

RP: reference panel

MFI: median fluorescence intensity

^1^ cut-offs determined by optimizing specificity and sensitivity of overall HHV species seropositivity

### HSV species-specific Monoplex Serology validation

Two antigens each were evaluated for their ability to determine HSV species-specific serostatus, HSV-1 gG and gD, and HSV-2 mgGunique (mgGu) and gD. Evolutionary, the HSV glycoproteins gG evolved differently resulting in an approximately 500 aa region unique to HSV-2 [38]. The sequence identity of the regions common to both HSV species is approximately 50% [38]. For HSV-2, gG is cleaved into a secreted (sgG) and a membrane anchored part (mgG) [38] of which the region unique to HSV-2 (mgGu) was expressed and used as antigen in HSV-2 Monoplex Serology. For HSV-1 and -2 gD, sequence identity is approximately 80%. Thus, antibody responses against gD antigens are of high interest for assessing cross-reactivity between both assays.

HSV-1 and -2 Monoplex Serology assays were validated against a HSV species-unspecific (Enzygnost anti-HSV IgG), and a HSV-2 specific (LIAISON HSV-2 IgG) reference assay in RP I and II, respectively. No HSV-1 species-specific reference panel was available. Thus, an indirect approach using HSV-2 Monoplex Serology validation was pursued for HSV-1 Monoplex Serology validation.

The measured antibody reactivities against HSV-1 and -2 antigens based on RP I are shown in Fig 1-I. Both HSV-1 antigens gD and gG discriminate well between reference assay positives and negatives, resulting in only one false-positive and 5 and 3 false-negatives, respectively. The resulting specificity is 98.8% for both HSV-1 antigens, while the sensitivity is 95.9% for gD and 97.6% for gG (Table 3). Based on RP I, HSV-2 gD also showed good capacity to distinguish between reference assay negatives and positives. However, in comparison to HSV-1 gD, a slightly larger overlap in measured antibody reactivities between reference assay negatives and positives was observed between approximately 10 and 100 MFI. HSV-2 mgGu showed very little seroreactivity in RP I, with only six sera showing antibody responses >100 MFI. Thus, no attempt was made to determine meaningful cut-offs for HSV-2 antigens in RP I.

HSV-2 Monoplex Serology was validated against a HSV-2-specific reference assay based on RP II (Fig 1-II). With a cut-off of 180 MFI, HSV-2 mgGu discriminated very well between reference assay positives and negatives resulting in only one false-positive and 4 false-negatives, yielding a specificity and sensitivity of 97.8% and 93.4%, respectively. *Kappa* statistics showed almost perfect agreement with the reference assay (*k* = 0.91) (Table 3). For reference assay positives, similar antibody reactivities were observed for HSV-2 gD and mgGu (approx. 200 to 10,000 MFI). However, high HSV-2 gD antibody reactivities were also observed for a substantial number of reference assay negative samples that were also negative for HSV-2 mgGu. Thus, no cut-off for HSV-2 gD was determined in RP II.

HSV-2 Monoplex Serology was successfully validated based on antigen mgGu. Based on the very low prevalence of HSV-2 mgGu, RP I contained very few (<5%) HSV-2 seropositives. Thus, the antibody reactivities measured with HSV-2 gD most likely represent cross-reactivity with HSV-1 gD antibodies. This was confirmed by a high correlation of antibody reactivities between the homologous HSV-1 and -2 gD proteins observed in both RP I and RP II (S1 Fig). Thus, the gD antigens could not be validated for measurement of species-specific HSV antibodies but can be applied for detection of general (species-unspecific) HSV infection. Additionally, based on the small number of HSV-2 seropositives in RP I, this setting allowed us to indirectly validate HSV-1 Monoplex Serology based on antigen gG (RP I) although no HSV-1 species-specific reference panel was available. This is further supported by the low correlation of gG and mgGu (r = 0.13, S2 Fig).

### VZV Monoplex Serology validation

The VZV Monoplex Serology antigen panel included glycoproteins E (gE) and I (gI) and immediate early protein 63 (IE63) and was validated against the time resolved fluorescence immunoassay (TRFIA) [31]. Antigen gE differentiated well between reference assay negatives and positives, with only 4 false-positives and 9 false-negatives (Fig 1-III); specificity was 95.2% and sensitivity was 90.7% (Table 3). *Kappa* statistics showed almost perfect agreement with the reference assay (*k* = 0.86). gI Monoplex Serology resulted in a substantial number of false-positives and even more false-negatives compared to the reference assay (Fig 1-III). Using a cut-off to yield the minimum desired specificity of 85.0%, calculated specificity was 89.2% and sensitivity was 58.8% (Table 3). Antigen IE63 showed no capacity to discriminate between reference assay negative and positive sera (Fig 1-III). Thus, IE63 is not informative for VZV Monoplex Serology and no cut-off was determined.

VZV proteins gE and gI form hetero-dimers in infected cells [39]. Thus, different approaches were undertaken to assess whether individual antigen performance of gE can be improved by a combination with gI. Determination of overall VZV serostatus by seropositivity to gE and / or gI resulted in very similar statistics compared to gE alone (Tables 3 and 4). To simulate hetero-dimerisation of gE and gI, two additional strategies were pursued to enable detection of VZV antibodies directed against epitopes jointly formed by gE and gI. First, bacterial lysates containing VZV antigens gE and gI were mixed and simultaneously loaded onto the same bead set. Co-loading of gE and gI showed very similar detection characteristics as gE individually (Fig 1-III and Table 3). However, antibody reactivities for both reference assay negatives and positives were slightly increased. In a second approach, antigens gE and gI were co-expressed in *E*. *coli* yielding almost identical data compared to co-loading, or gE alone (S3 Fig).

Thus, VZV Monoplex Serology performance is largely driven by antigen gE. In RP III, no added benefit could be achieved by the various approaches to include gI.

### EBV Monoplex Serology validation

EBV Monoplex Serology comprises a panel of four EBV proteins. In case of EBV nuclear antigen 1 (EBNA-1), two fragments of differing sizes were expressed, EBNA-1 truncated (EBNA-1 trunc) and EBNA-1 peptide (EBNA-1 pep) (Table 1). In addition, viral capsid antigen p18 (VCA p18), Z-Epstein-Barr virus replication activator (ZEBRA) and early antigen-diffuse (EA-D) were included in the EBV antigen panel. EBV Monoplex Serology was validated against the Enzygnost anti-EBV IgG assay. Among the reference assay seropositive sera (n = 136), 131 (96.3%) were seropositive for EBNA-1 trunc, 124 (91.2%) for VCA p18, 117 (86.0%) for EBNA-1 peptide, 110 (80.9%) for ZEBRA and 103 (75.7%) for EA-D (according to cut-offs shown in Table 4). Antigen-specific concordance with the reference assay is generally good (Fig 1-IV). However, for all antigens between 5 and 22 false-positives and/or false-negatives were observed. Specificity for individual antigens ranged between 83.1% for EA-D and 92.3% for both EBNA-1 trunc and VCA p18 (Table 3). Sensitivity is very similar, and between 83.8% for EA-D and 96.3% for EBNA-1 trunc. *Kappa* statistics showed substantial agreements for EBNA-1 pep, ZEBRA and EA-D and almost perfect agreement for VCA p18 and EBNA-1 trunc (*k* = 0.64 to *k* = 0.89).

Only 2 of the reference assay seropositive sera did not react with any or only one of the EBV antigens; 86 (63.2%) were seropositive against all four EBV antigens (EBNA-1 trunc, VCA p18, ZEBRA, EA-D), 29 (21.3%) against 3 and 17 (12.5%) against 2. Nine reference assay negative sera showed antibody responses against at least one antigen; in 5 of these, antibodies against multiple EBV antigens were detected. Determining overall EBV seropositivity by a combination of the antigens showed optimum specificity (92.3%) and sensitivity (97.1%) by seropositivity against at least 2 out of 4 EBV proteins (Table 4). Using this algorithm, almost perfect agreement between both EBV assays (*k* = 0.90) was reached. Inclusion of either EBNA-1 trunc or peptide in the algorithm showed to be equally specific, but slightly more sensitive when including EBNA-1 trunc (Table 4).

### CMV Monoplex Serology validation

CMV Monoplex Serology is based on four proteins: pp52, pp28, pp65 and pp150 N-terminus (pp150N) and was validated against two different reference assays, an anti-CMV IgG ELISA based on commercially available virion proteins (RP Va) and the Enzygnost anti-CMV IgG (RP Vb). Antibody detection against individual CMV antigens pp28, pp52 and pp150N showed high concordance with both reference assays detecting a maximum of 6 false-positives or false-negatives (Fig 1-Va/Vb). Specificity ranged between 95.4% and 99.0% and sensitivity ranged between 94.0% and 98.7% (Table 3). *Kappa* statistics indicated almost perfect agreement with both reference assays (*kappa* 0.90 to 0.96). Assay performance for CMV antigen pp65 was slightly poorer in comparison with the other antigens showing a higher overlap of measured antibody reactivities for reference assay positive and negative samples in both reference panels (Fig 1-Va/Vb). However, *kappa* statistics still indicated almost perfect agreement with both reference assays (Va, *k* = 0.83; Vb, *k* = 0.85) (Table 3).

Overall CMV serostatus was determined by seropositivity against at least two out of three CMV antigens (pp28, pp52, pp150N) as inclusion of pp65 did not additionally improve assay performance and is therefore dispensable. Specificity was 96.9% and 100.0% for the two reference panels, while sensitivity was 98.7% and 96.0%, respectively (Table 4).

### Comparison of performance of HHV Monoplex and Multiplex Serology

**Fig 2:**
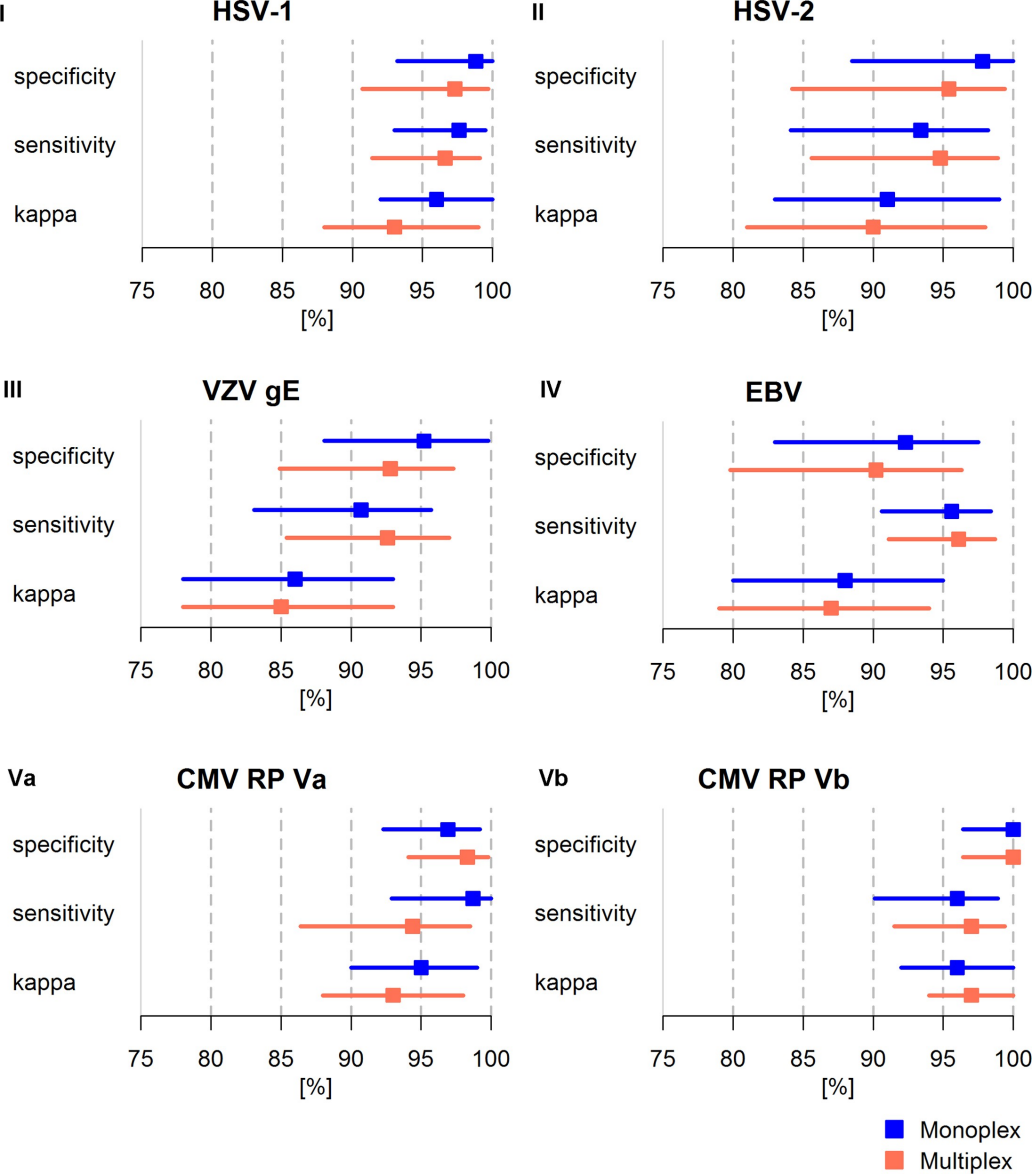
Comparison of statistical performance of HHV species-specific assays in monoplex (blue) and multiplex (orange) format. Performance is shown for overall seropositivity (EBV, CMV) and single antigens for HSV-1 (gG), HSV-2 (mgGu) and VZV (gE). I-Vb indicate corresponding reference panels. Cohen’s *kappa* statistics are shown in percent to improve visualization. For direct comparison of Monoplex and Multiplex Serology performance on the corresponding reference panel, ICCs were calculated showing good to excellent reliability (ICC_HSV1_: 0.91 (95% CI 0.88–0.93), ICC_HSV2_: 0.93 (95% CI 0.89–0.95), ICC_VZV_: 0.93 (95% CI 0.91–0.95), ICC_EBV_: 0.87 (95% CI 0.84–0.90), ICC_CMV RPVa_: 0.82 (95% CI 0.77–0.86), ICC_CMV RPVb_: 0.99 (95%CI 0.99–1.00)). kappa: Cohen’s kappa. ICC: Intraclass correlation coefficient. CI: confidence interval.

The reference sera were analyzed both in monoplex (i.e. one pathogen) and in multiplex (i.e. multiple pathogens) format in order to compare assay performance. The performance of HHV Multiplex Serology based on sensitivity, specificity and *kappa* statistics was evaluated in comparison to the corresponding species-specific Monoplex Serology results (Fig 2). While some of the individual species-specific HHV assays showed slight differences in sensitivity and specificity between the monoplex and multiplex format, the overall statistical performance of the species-specific assays in Multiplex Serology did not change. Sensitivity and specificity for overall seropositivity for all HHV species exceeded 90% in both monoplex and multiplex format. A high concordance with the corresponding reference assays was maintained (*k* ≥ 0.85). A direct comparison of Monoplex versus Multiplex Serology performance was conducted using ICCs and showed good to excellent reliability (ICC: 0.82–0.99).

## Discussion

We report the development and successful validation of HHV Monoplex Serology assays against 5 out of 9 known human herpesviruses, namely HSV-1, HSV-2, VZV, EBV and CMV. The species-specific Monoplex Serology assays were validated using reference sera analyzed with gold-standard reference assays. For each species-specific assay, only a small number of false-positives and false-negatives was observed during validation. This resulted in a median specificity and sensitivity of 97.4% and 96.6%, respectively. The newly developed assays were found to be highly concordant with the established gold-standard reference assays (median *kappa* 0.93). Robust statistical assay characteristics of HHV species-specific serological assays were confirmed in a multiplex setting comprising a larger antigen panel including multiple additional pathogen-specific Monoplex Serology assays (Fig 2).

A minimum number of antigens per species-specific assay was pursued to facilitate the incorporation into larger Multiplex Serology panels comprising additional infectious disease antigens. By selecting a total of 10 antigens for the first 5 HHVs, this has been [e.g. [40, 41, 42, 43] and will be feasible in future seroepidemiological studies.

HSV glycoproteins gG are the ideal candidate antigens in serological assays aiming at HSV species-specificity due to reported low sequence identity [38], and low observed correlation of antibody responses in Multiplex Serology (S2 Fig). Thus, the evaluated HSV gG antigens most likely allow to measure species-specific antibody reactivities, while HSV-1 and 2 antigens gD detect non-species-specific HSV infection confirming previous reports [38, 44]. HSV-1 Monoplex Serology was indirectly validated against the HSV non-species-specific Enzygnost anti-HSV IgG assay. The assay was reported to be based on crude lysate of HSV-1 infected cells [45]. Although HSV-1 Monoplex Serology is based on one antigen (gG) only, assay concordance is almost perfect (*k* = 0.96). This implies that missing glycosylation due to recombinant antigen expression in *E*. *coli* does not seem to impair the immunogenicity of the epitopes.

HSV-2 Monoplex Serology was successfully validated against the LIAISON HSV-2 IgG chemiluminescent immunoassay based on recombinant HSV-2 gG antigen [46]. As both the HSV-2 Monoplex Serology and the reference assay are based on the same HSV-2 protein, high concordance (*k* = 0.91) is not surprising. Only five discordantly classified samples were observed, potentially due to different expression systems or diverging antigen sequences. Sequencing of HSV-2 antigen mgGu revealed 3 deviations from HSV-2 strain HG52 on the protein sequence level: an amino acid exchange from Threonine to Alanine, a single amino acid deletion and a 14-amino acid deletion. This might indicate mutations inserted during PCR, errors during sequencing, or deviations in the parental DNA. In fact, our recombinant antigen is based on an untyped genomic DNA potentially representing a clinical isolate, or a different strain. Despite the detected potential sequence deviations, high concordance with the reference assay was reached. Thus, these deviations most likely do not result in conformational changes within immunogenic epitopes of HSV-2.

In reference panel I, 4 sera with HSV-2 mgGu antibody responses > 180 MFI were detected. These were also seropositive against HSV-1 gG. Based on the low correlation between HSV-2 mgGu and HSV-1 gG (S2 Fig), we assume that these 4 seropositive individuals were co-infected by HSV-1 and HSV-2. This is further supported by the high HSV-1 prevalence in the general population and their shared route of exposure [6, 7]. However, these 4 individuals could also represent HSV-2 infected individuals with antibodies against the non-unique epitopes of HSV-2 gG cross-reacting with HSV-1 gG.

EBV Monoplex Serology was successfully validated against the Enzygnost anti-EBV IgG assay showing high concordance for overall EBV seropositivity (*k* = 0.88 and *k* = 0.90, depending on the EBNA-1 antigen). The reference assay was reported to be based on a mixture of EBV VCA, EBNA and EA antigens. The exact composition is unknown to the authors of this paper. However, the usage of probably overlapping antigen panels in EBV Monoplex Serology and the reference assay likely explains high concordance.

EBV Monoplex Serology uses antigens expressed during different stages of the EBV life cycle. While EBNA-1 is expressed during latent infection, ZEBRA, EA-D and VCA p18 are expressed during the lytic stage [17, 47]. Detection of antibodies to EBV VCA and EBNA-1 were reported to distinguish EBV infection history; IgG and IgM antibodies against EBV VCA proteins mark acute infection, while presence of only IgG antibodies against VCA and EBNA-1 serologically defines past infection or late primary infection. This pattern in combination with IgM antibodies against VCA proteins also marks reactivation [47]. As a viral capsid protein, VCA p18 has expectedly been presented to the immune system of all EBV infected individuals upon primary infection. Thus, detection of 124 (91.2%) reference seropositive sera with VCAp18 antibody reactivities above the cut-off is consistent with its potential role as marker for acute and past infection. Among the reference assay seropositive sera, 109 (80.1%) and 121 (88.8%) sera were seropositive against VCA p18 and EBNA-1 pep or EBNA-1 trunc, respectively. Thus, these represent most likely past primary and latent infection. However, of the 124 VCA p18 seropositive sera, 110 were seropositive for either EA-D or ZEBRA, or both. High antibody reactivities against these antigens might represent markers of previous reactivation as they are expressed early in the lytic stage and EA-D IgG antibodies were reported to be detectable only temporarily after the lytic phase of EBV infection [47]. Four out of 5 detected false-positive sera were seropositive against VCA p18 plus at least two other EBV antigens. This raises the question whether these sera are likely to be true EBV positives, and whether EBV Monoplex Serology might be slightly more sensitive than the reference assay.

Recently, Coghill *et al*. reported a risk stratification signature for nasopharyngeal carcinoma in Taiwan based on EBV IgG and IgA antibodies [48]. The possible detection of antibody patterns specific for past versus reactivated EBV infection, in combination with the findings by Coghill *et al*. imply the potential for future disease-specific EBV antibody patterns in Multiplex Serology based on separate measurements of IgG, IgM and IgA.

EBV elicits high antibody responses in infected individuals. For at least semi-quantitative measurement of antibodies within the dynamic range of the assay, serum dilutions must align with the expected antibody titers elicited by the pathogen. Thus, antibody measurements at high serum dilutions are recommended for EBV. Validation was performed at dilution 1:1000, i.e. the standard dilution of Multiplex Serology for the simultaneous measurement of antibodies against many pathogens. Inclusion of two EBNA-1 antigens of differential length in the algorithm defining overall EBV seropositivity did result in slightly higher sensitivity for EBNA-1 trunc. However, antibody measurements against the peptide show a wider dynamic range (Fig 1-IV). Thus, antigen EBNA-1 peptide instead of EBNA-1 trunc enables more quantitative antibody measurements at dilution 1:1000 with only marginally reduced assay sensitivity.

VZV Monoplex Serology based on antigens gE and gI was validated against the TRFIA developed by McDonald *et al*. [49]. The TRFIA is based on a sucrose density gradient centrifugation-purified extract of human embryo lung-cultured VZV strain Ellen detecting anti-VZV IgG [31]. Observed false-negative sera might not react with the antigens gE or gI, but another antigen of the VZV proteome present in the TRFIA. Sequences for expressed gE and gI antigens (strain Dumas) were compared with strain Ellen (reference assay) and were found to be highly concordant (min. 99%).

For the VZV TRFIA, 84% agreement with the Fluorescence Antibody to Membrane Antigen (FAMA) assay was reported [49]. Although we did not directly compare our VZV Monoplex Serology assay to the VZV FAMA assay, specificity and sensitivity >90% in comparison to the TRFIA indirectly confirm substantial agreement with the FAMA assay.

CMV Monoplex Serology performed very well in comparison with two independent reference assays, the Enzygnost anti-CMV IgG and the CMV ELISA developed by the Stanley Neurovirology Laboratory [34]. The Enzygnost assay was reported to be based on inactivated antigens from CMV infected human fibroblasts, while the second CMV reference assay was reported to use purified CMV antigen [34, 50, 51, 52]. The exact antigen compositions of both CMV reference assays are thus unknown to the authors of this paper. Despite potentially different antigen composition used in the reference assays, very good agreement of CMV Monoplex Serology with the two independent reference assays was observed. Additionally, the reported validation of the Enzygnost assay against a CMV IgG assay on the Abbott Architect platform [53] confirms robust and efficient detection of CMV infection with CMV Monoplex Serology.

CMV Monoplex Serology was applied to two different reference serum panels. Depending on the reference panel and corresponding reference assay, different cut-offs were found to optimize statistical characteristics per antigen. Thus, we conclude that cut-offs might not be directly transferable between studies. This might have multiple potential reasons such as differences in the underlying study population, differential blood collection conditions and storage of serum specimens before testing, as well as potential assay drift and reagent performance over time. This can be accounted for by standard quality control and normalization procedures between studies. In addition, differential underlying reference assay characteristics cannot be excluded by only pair-wise comparison of CMV Monoplex Serology with each reference assay, and might have influenced the selected optimum cut-offs.

Similarly to the above described assays, antigens for species-specific Monoplex Serology assays were developed for human herpesviruses HHV-6A & B, HHV-7 and KSHV. To our knowledge, there are no universally applicable serological gold standard assays with sufficiently acceptable performance characteristics for clinical use available for these HHV species. Thus, the developed Monoplex Serology assays could not be validated so far.

Multiplex Serology uses a secondary antibody directed against human IgG, IgM and IgA antibodies. However, this set-up does not allow the discrimination between acute infection during which IgM antibodies are detectable and past infection marked by IgG antibodies. To allow for specific detection of IgM antibodies, the HHV Monoplex Serology assays could be adapted and re-validated using a secondary antibody detecting human IgM, and corresponding reference panels.

Herpesviruses utilize various mechanisms to interact with their hosts that may lead to cancer initiation and progression. All HHV species have been associated with different types of cancer [10]. However, except for EBV and KSHV, scientific evidence for a role of herpesviruses in cancer development is controversially discussed [10, 54]. In addition, human herpesviruses have been associated with coronary heart disease (HSV-1, VZV, EBV, CMV) and neurodegenerative diseases such as Alzheimer’s disease (HSV-1) and multiple sclerosis (HHV-6, EBV) [55, 56, 57, 58, 59, 60, 61, 62, 63, 64]. Large prospective cohort studies provide not only the statistical power but also a suitable study design (i.e., pre-diagnostic exposure assessment) to study the role of human herpesviruses in disease etiology. Such large studies require cost-efficient methods to detect past or present viral infections, with minimal sample volume requirements. The developed and validated HHV Multiplex Serology enables simultaneous and efficient detection of infections by human herpesviruses 1 to 5.

## Supporting information

S1 Fig Scatter plot of HSV-1 gD and HSV-2 gD antibody reactivities (MFI) in reference panels I (I) and II (II) grouped by reference assay serostatus. In both cases, Pearson’s r is 0.68.
Refstat: reference assay serostatus.
MFI: Median Fluorescence Intensity.
(TIF)

S2 Fig Scatter plot of HSV-1 gG and HSV-2 mgGu antibody reactivities in reference panel II. For most sera, no correlation of antibody reactivities against HSV-1 gG and HSV-2 mgGu was observed. Some sera were reactive against both HSV-1 gG and HSV-2 mgGu most probably representing co-infection of HSV-1 and HSV-2 instead of cross-reactivity.
MFI: Median Fluorescence Intensity.
(TIFF)

S3 Fig Comparison of quantitative antibody measurements (MFI) against antigen gE, co-loading of gE and gI (gE/gI) and co-expression of gE and gI (Coexpr) stratified by VZV reference serostatus. Sera from RP III were tested at serum dilution 1:1000.
gE/gI: co-loading of antigens gE and gI.
Coexpr: co-expression of antigens gE and gI.
(TIFF)
