# Supplementary figures and images for: Validation of Multiplex Serology detecting human herpesviruses 1-5
Source: PLoS One. 2018 Dec 27;13(12):e0209379. doi: 10.1371/journal.pone.0209379 (PMC6307738; doi:10.1371/journal.pone.0209379)

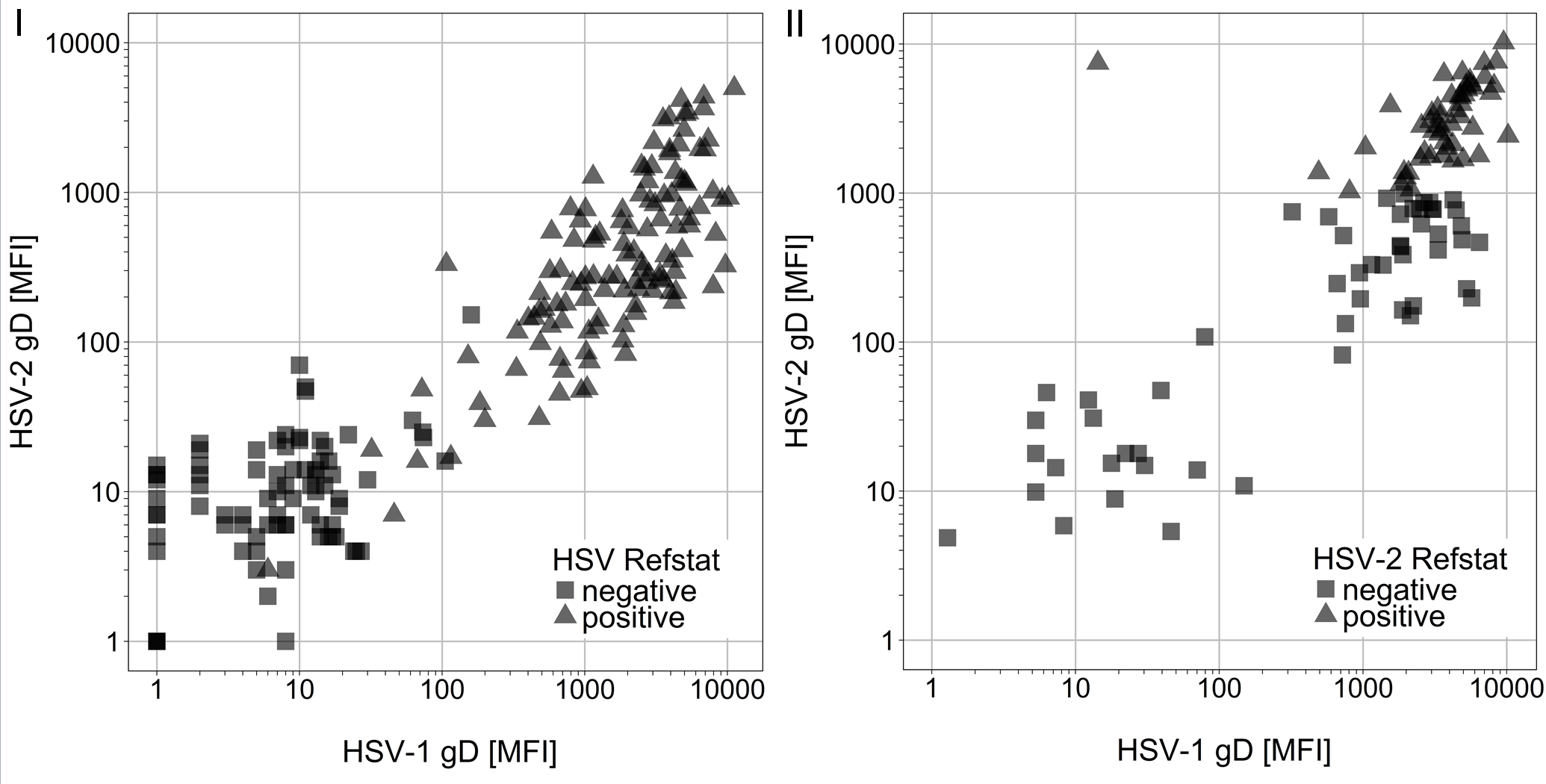

Supplement: S1 Fig — In both cases, Pearson’s r is 0.68. Refstat: reference assay serostatus. MFI: Median Fluorescence Intensity. (TIF) [file pone.0209379.s001.tif]

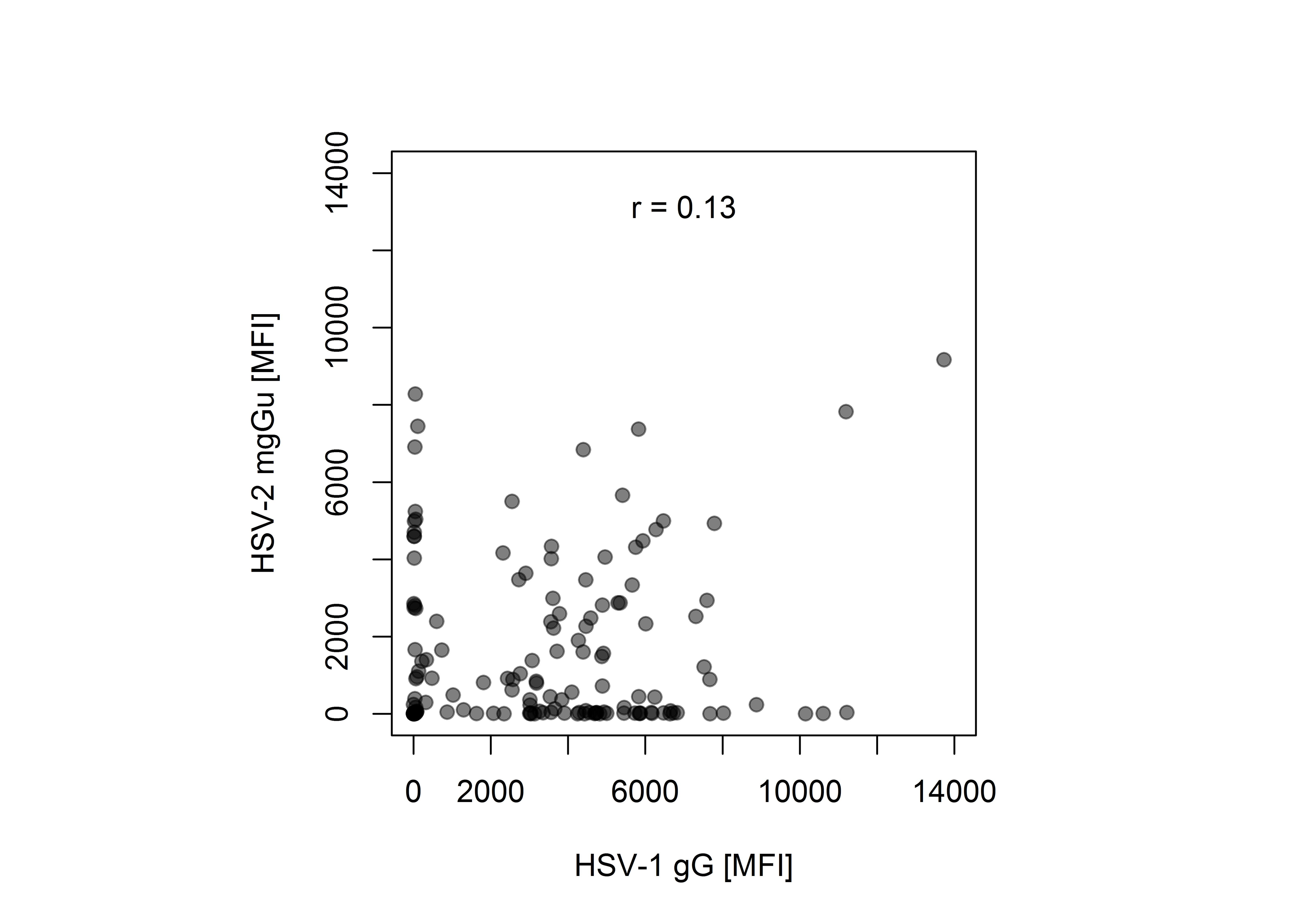

Supplement: S2 Fig — For most sera, no correlation of antibody reactivities against HSV-1 gG and HSV-2 mgGu was observed. Some sera were reactive against both HSV-1 gG and HSV-2 mgGu most probably representing co-infection of HSV-1 and HSV-2 instead of cross-reactivity. MFI: Median Fluorescence Intensity. (TIFF) [file pone.0209379.s002.tiff]

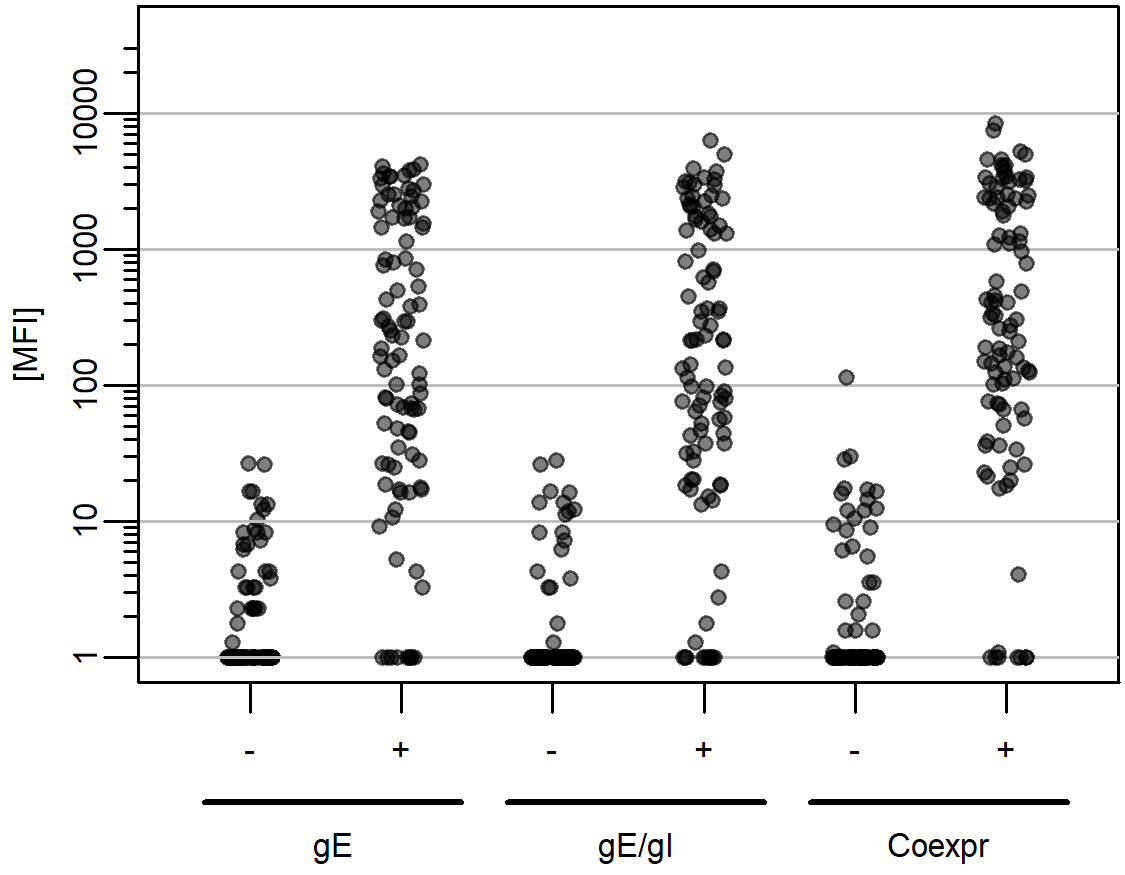

Supplement: S3 Fig — Sera from RP III were tested at serum dilution 1:1000. gE/gI: co-loading of antigens gE and gI. Coexpr: co-expression of antigens gE and gI. (TIFF) [file pone.0209379.s003.tiff]
